# Barriers and facilitators of the reporting by family doctors of cases of domestic violence – a qualitative study across Portugal

**DOI:** 10.1186/s12875-024-02329-0

**Published:** 2024-04-05

**Authors:** Diana Nadine Moreira, Mariana Pinto da Costa

**Affiliations:** 1 Institute of Biomedical Sciences Abel Salazar (ICBAS), University of Porto, Rua Jorge de Viterbo Ferreira, nº 228, Porto, 4050-313 Portugal; 2 Institute of Public Health of the University of Porto, Porto, Portugal; 3 Institute of Psychiatry, Psychology & Neuroscience, King’s College London, 16 De Crespigny Park, London, SE5 8AB UK

**Keywords:** Domestic violence, Family medicine, General Practitioners, Mandatory reporting, Qualitative study, Portugal

## Abstract

**Background:**

Domestic violence (DV) is a serious and prevalent public health problem with devastating consequences for the victims and their families. Whilst the number of cases reported to the authorities has risen in recent years, many victims still chose not to present a complaint. In Portugal, to address this, DV became a public crime. As victims of DV present multiple health problems and frequently seek professional help, family doctors are in a privileged position to detect and report cases of DV to the authorities. However, little is known about what motivates these professionals to report or not the DV cases they encounter in their practice to the authorities.

**Methods:**

We conducted semi-structured interviews with family doctors from all regional health administrations of continental Portugal. Interviews occurred between July 2020 and September 2022, were conducted in person or remotely, audio recorded, transcribed, and analysed using thematic analysis. Content analysis was conducted to assess the agreement or disagreement regarding mandatory reporting in each of the themes and subthemes.

**Results:**

Fifty-four family doctors took part in this study (*n* = 39 women, *n* = 15 men). The main themes that arose from the analysis were: “Barriers related to the physician’s activity,” “Barriers related to the victim or aggressor,” “Facilitators related to the physician’s activity,” “Facilitators related to the victim or aggressor.” Although different barriers were described, most doctors agreed with the mandatory reporting of DV cases.

**Conclusions:**

Family doctors encounter multiple barriers and facilitators when considering reporting a DV case to the authorities. The results of this study can help develop new interventions to address the barriers described by the doctors, increasing their compliance with mandatory reporting, the protection of victims and the just persecution of the aggressor.

**Supplementary Information:**

The online version contains supplementary material available at 10.1186/s12875-024-02329-0.

## Background

Domestic violence (DV) is a serious social and public health problem, highly prevalent and globally widespread [1]. It encompasses any act or threat of physical, psychological, or sexual violence perpetrated within the context of an intimate relationship [2]. The World Health Organization estimates that one in every three women experiences physical and/or sexual violence during their lifetime [3]. Among male victims’ the prevalence varies between one in every four to one in every ten men, according to different studies [4, 5].

In Portugal, DV is the most frequently committed crime [6]. During the first semester of 2022, the Commission for Citizenship and Gender Equality based on the numbers provided by the Public Security Police (PSP) and the Republican National Guard (GNR) registered 14363 DV reports to those law enforcement entities, which correspond to 2192 more cases compared with the same period in 2021 [7]. It is thought that most cases of DV are never reported. The Portuguese Support Victims Association (APAV) assists victims of multiple crimes. From the 25838 support requests presented to APAV in 2021, 76.8% (*n* = 19846) were related to DV. However, only 46% (*n* = 6067) of the total of request were reported to the police [8].

To address the number of reports, provide victims protection, and ensure the persecution of the aggressors, DV became a public crime in Portugal in 2000. This means that anyone can file a DV report, which is enough to progress the criminal proceedings, even against the victim’s will. The facultative character of the report applies to the general population but in the case of public workers, such as policemen and health care professionals it is a professional obligation. These mandatory reporting legislations have been regarded as controversial policies, with limited studies to support their efficiency and applicability [9].

Family doctors provide continuous follow-up of their patients and families, sometimes through several years, developing a close relationship with them. The characteristics of family medicine place family doctors in a particularly privileged position to identify, refer, and report DV cases they may encounter in their clinical practice. DV victims’ resort to health care services more frequently than the general population presenting multiple health conditions as a result of acute or chronic abuse [10–12]. These contacts with the health system provide important opportunities for intervention, and according to mandatory reporting legislations, these should result in a report to the authorities. However, little is known regarding the degree of compliance with the law by family doctors and what motivates them to present, or not, a report to the authorities.

Therefore this study aimed: i) to assess the barriers and facilitators that make family doctors in Portugal to present, or not, a report to the authorities of a DV case they encounter in their clinical practice, and ii) to assess the agreement or disagreement of family doctors in Portugal with mandatory reporting of DV in association with the factors influencing their decision to present or not a report.

## Methods

### Study design

We conducted a qualitative study based on semi-structured interviews with family doctors in Portugal. The interview guide was developed by the authors. Prior to the interview, participants’ sociodemographic data was collected using an online questionnaire.

### Setting and participants recruitment

This study was conducted in the five Regional Health Administrations (RHA) of continental Portugal: North, Center, Lisbon and Tagus Valley, Alentejo and Algarve. An email was sent to every Family Health Unit and Personalized Health Care Unit of each of the RHAs. This email introduced the study, inviting family doctors of each institution to take part. It also contained the email address of the lead author (DNM) and a link that would direct the potential participants to an online page containing the information sheet with additional detailed information about the project, the informed consent and the sociodemographic questionnaire. This page was also promoted in several social media groups and online forums for family medicine doctors. At the end of the online questionnaire, it was requested the participant’s email to enable further contact and the scheduling of the interview. The interviews were conducted by the lead author (DNM) through online platforms: Zoom, Skype, or Teams, or in person when possible. The only inclusion criteria was to be a specialist in family medicine currently working in continental Portugal.

### Data collection and analysis

The interviews took place between July 2020 and September 2022, and were conducted according to an interview guide developed by the authors. Interviews were audio recorded, transcribed, and analysed through thematic analysis using the approach proposed by Braun & Clarke [13]. Transcripts were uploaded into QSR International Nvivo version 12, which was used to manage and analyse the data. The analysis was inductive and based on the content of the transcripts rather than on any existing theory or hypothesis. Themes and subthemes were reviewed independently by both authors several times during the analysis process to guarantee internal consistency between their conceptual nucleus and the codes that generated them. Coding saturation was achieved and no new codes emerged from the data. The heterogeneity of the population studied and the broad research question justify the need for a large number of interviews to reach coding saturation. Content analysis was used to assess which theme and subthemes were linked to a favourable or unfavourable position of participants regarding mandatory reporting of domestic violence.

## Results

Eighty-four doctors responded to the online questionnaire, after which they were contacted via email by the lead author (DNM). Of these, two changed their mind and declined to be interviewed, two scheduled the interview but did not show up, and twenty-six never replied to the email to schedule the interview. In total 54 participants were interviewed (12 from the North RHA, 12 from the Center RHA, 12 from the Lisbon and Tagus Valley RHA, 6 from the Alentejo RHA, and 12 from the Algarve RHA). Six participants knew the lead author previously to the interview. The median duration for an interview was 23 min (IQR 9:00 to 45:00 min).

The majority of participants were female (72%) and their age ranged from 30 to 65 years. The detailed socio-demographic information of the participants is presented in Table 1.

**Table 1:** Participants socio-demographic characteristics (*N*=54)

	**Characteristic**	**(*N, %* )**
**Gender**	Female	39 (72)
	Male	15 (28)
**Age**	25–34 years	15 (28)
	35–44 years	21 (39)
	45–54 years	9 (17)
	55–64 years	8 (15)
	> 65 years	1 (2)
**Sexual orientation**	Heterosexual	50 (93)
	Homosexual	1 (2)
	Bisexual	2 (4)
	NR	1 (2)
**Ethnicity**	White	50 (93)
	Black	1 (2)
	Mixed ethnicity	2 (4)
	NR	1 (2)
**Marital status**	Single	7 (13)
	In a relationship without cohabitation	1 (2)
	In a relationship with cohabitation	12 (22)
	Married	28 (52)
	Divorced	5 (9)
	NR	1 (2)
**Years of professional experience**	1–9 years	24 (44)
	10–19 years	17 (32)
	20–29 years	4 (7)
	30–39 years	9 (17)

*NR*: no response

**Fig. 1:**
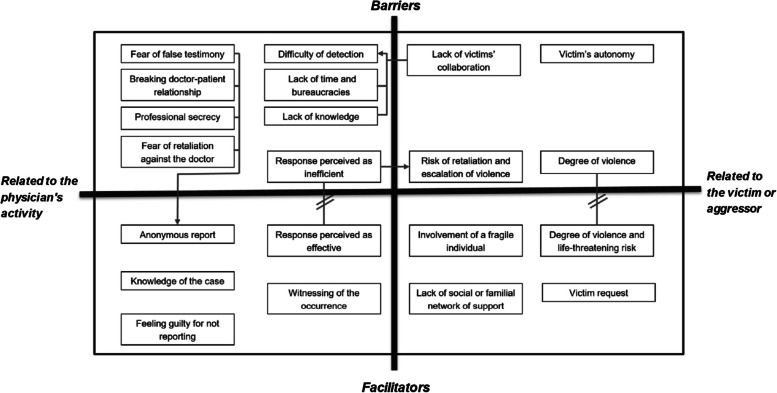
Relations between the themes and subthemes

The data was organized into four broad themes, each with several sub-themes: 1) “Barriers related to the physician’s activity”; 2) “Barriers related to the victim or aggressor”; 3) “Facilitators related to the physician’s activity”; 4) “Facilitators related to the victim or aggressor” (Table 2). These barriers and facilitators are frequently interconnected representing the complexity of DV cases (Fig. 1: Relations between the themes and subthemes).

**Table 2:** Themes and subthemes

***Themes:***			
***Barriers related to the physician’s activity***	***Barriers related to the victim or aggressor***	***Facilitators related to the physician’s activity***	***Facilitators related to the victim or aggressor***
***Subthemes:***			
Difficulty of detection Lack of time and bureaucracies Lack of knowledge Lack of guidelines Fear of false testimony Breaking doctor-patient relationship Professional secrecy Fear of retaliation against the doctor Alternatives perceived as more beneficial Response perceived as inefficient Not being a doctor’s responsibility Violence as an acceptable response to violence	Victims autonomy Degree of violence Risk of retaliation and escalation of violence Lack of victims’ collaboration	Anonymous report Knowledge of the case Feeling guilty for not reporting Response perceived as effective Witnessing of the occurrence	Involvement of a fragile individual Lack of social or familial network of support Victim request Degree of violence and life-threatening risk

### Barriers related to the physician’s activity

Multiple barriers were described with relation to the reporting of domestic violence cases to the authorities that were linked to the doctors’ clinical activity, their fears, and perceptions. One of the first barriers described was the difficulty of detection. In the absence of physical evidence, and when the patient purposely tries to hide, or disguise the causes of their afflictions, doctors’ ability to detect domestic violence cases can be compromised. The interviewed doctors made reference to the multiple signs and symptoms, such as headaches, abdominal pain, and nocturnal enuresis in children that can be related to DV. When the doctor fails to make the connection between these health problems and a possible case of abuse, they may lead to multiple consultations, exams and treatments that fail to address the underlying issue.

*“I can’t always understand, sometimes I may suspect something, but if the patient doesn’t tell me anything, does not confirm my suspicion, I’m not always able, and don’t have the time, to pay attention to all the signs, and remember “Wait, this could be domestic violence.””* (Participant 26, Woman)

The difficulty of detection was frequently associated with other barriers such as lack of time and bureaucracies. Several doctors complained about the lack of time in their clinical practice to address DV concerns. They spoke about having a limited time for each consultation, the pressure felt in the waiting room when there is a delay, the high number of patients under their care, and the fact that in most consultations they are expected to address multiple issues and health problems. Doctors also said they did not have the time to deal with the possible increase in bureaucratic paperwork associated with a complaint to the authorities.

*“I also want to say that in family medicine we have it all. What we don’t have is the necessary time to deal with all of this. Because there is an excess of bureaucratic work.”* (Participant 10, Man)

However, they also express not knowing in reality which bureaucracies and procedures would be implicated. Lack of knowledge was the main barrier to the reporting of DV cases described by the doctors. Some doctors admitted to being uninformed, lacking the knowledge and capabilities to manage, refer, and report a DV case.

*“Honestly, I also don’t know exactly which are the procedures. (…) I have a victim here in front of me, who should I call? Honestly, it is a flaw on my part. I have to look into it later.”* (Participant 19, Man)

Other doctors, having recognized this gap in their knowledge, tried to look for information regarding DV, but were incorrectly or insufficiently enlightened.

*“You can’t. You can’t [referring to the reporting of DV cases]. I asked the juridic department. You can´t. I had that problem and you can’t. Because it is the story that that woman is telling you. She could have, she could have had an injury, she could… you don’t know. And since you don’t know, you didn’t see [the aggression], you can’t.”* (Participant 11, Woman)

One doctor described a case of a patient that complained multiple times to be a victim of DV perpetrated by her husband, presenting physical lesions compatible with the abuse described. This husband was known to be an aggressive individual, having assaulted a nurse and the security guard of the health centre. This doctor described that having sought advice from the juridical department of her workplace regarding the possibility of breaking professional confidentiality and reporting the abuse, she was misinformed that she could not report the case unless she was an eyewitness to the violence.

Other doctors described they were misinformed by their hierarchy superiors or by the police department when trying to present a report. This led doctors to request for training opportunities for health professionals and law enforcement regarding DV. Doctors also felt that there is a lack of guidelines regarding DV and reaffirmed the need for the creation and implementation of guidelines and procedures specific to each community.

*“There should be an algorithm, something concrete that would tell you, in this case, you must do this, refer to these and that institution, you can present a report using these and that means.”* (Participant 51, Woman)

Doctors frequently brought up the fact that they are in a particularly unique, but sometimes difficult position as doctors of the potential victim and aggressor. As family doctors, they are sometimes confronted with two different accounts of the same events, which in the absence of more concrete physical evidence may compromise their readiness to report.

*“Because it is like this, you rarely see any physical marks. Even a belt or something like that… it’s what people tell you (…), we don’t know what is true, we hear people talking, but we don’t know what is true, and then you have the other side of the story. Since we are a family doctor we have both components, and then sometimes as well… I’m not a cop! To clarify the situation. And I don’t always believe it.”* (Participant 39, Woman)

This fear of false testimony is frequently associated with other barriers such as the fear of breaking the doctor-patient relationship, either with the victim or the aggressor, especially if unjustly accused. Doctors impart great importance on their ability to establish a therapeutic relationship with their patients and fear that a report could break their trust compromising future interactions.

*“It is complicated to keep the relationship. Because you can never again establish a level of trust if you make a report. Who is gonna trust us? Neither the aggressor nor the victim. Especially the relationship with the aggressor is destroyed”.* (Participant 22, Woman)

A doctors-patient relationship is based on multiple assertions, one of which is the duty to keep the doctor’s patient confidentiality and professional secrecy. This secrecy is a right of the patient recognized by law and can be perceived as a conflicting concept with mandatory reporting.

*“Another question is doctor’s patient confidentiality, isn’t it? It all depends if the victim wants to present a report, and that isn’t a problem, right? Now if they inform me in confidence that they don’t want anyone to know, I have an obligation, by law, as a healthcare professional, to keep it secret. Then we enter a very sensitive area.”* (Participant 35, Woman)

Doctors also expressed concerns regarding the possible legal consequences of breaking doctor-patient confidentiality. This idea is included in another subtheme, fear of retaliation against the doctor. Doctors feared being reprimanded by their hierarchy superiors after a report, being sued for defamation, becoming a victim of physical violence by the aggressor, and seeing their reputation slander as an act of revenge. Many of the doctors interviewed cited that there is no security in their health unit feeling completely exposed and unsafe.

*“I explained everything [a suspected case of sexual violence of a father against his daughter stated in consultation by the mother] to the investigator, to the inspector, and he told me: “You know doctor, everyone says it’s not true, and right now you are their accuser. They can take you to court for defamation.” I was so choked I could die, as you can imagine*.” (Participant 06, Woman)

Doctors also considered that in some cases a report could be non-beneficial, giving priority to other kinds of interventions tailored to the victim and their situation. Doctors expressed that it could be more advantageous to the victim to be referred to support institutions like APAV or Adult Violence Preventions Teams (EPVA) so that the process of reporting the violence to the authorities could be worked out between them and the victims according to their wishes and understanding of their situation. Other suggested means of support mentioned were psychology and psychiatry consultations, referral to a social worker, and family therapy. These alternatives were perceived as more beneficial, especially in cases where the aggressor was violent as a result of a mental health disorder.

*“I think that in the case of this family reporting the case will not help, it will not be helpful for the police to go there, it’s not gonna help the family. Maybe as the family doctor, with the help of the psychologist, something more based on the family, maybe she [the victim] would gain conscience, and he [the aggressor] will realize that he is abusing her and that it is a crime, maybe if I say that and explained that his wife symptoms are related to the abuse, maybe that could help.”* (Participant 45, Woman)

Not only were alternatives sometimes perceived as more advantageous, but also, frequently, the results of reporting the case to the authorities were seen as subpar. The response provided after a report was considered inefficient in protecting the victims from possible retaliation from the aggressor and the punitive measures implemented to correct the aggressor’s behaviour were judged inefficient.

*“Is the woman really protected? He is punished in an exemplary fashion? How? In what way? Does he learn, does he not? (…) People told me: “Why would I report? The first time I went to the police and they told me: “Lady! For the love of God. We have better things to do! But then why? What is gonna happen? He isn’t going to jail! It’s no use to complain.””* (Participant 37, Man)

This discontent with the response offered after reporting mostly alluded to the police and juridical system. However, doctors also considered that sometimes the support provided by institutions such as APAV and EPVA was less than ideal.

*“I am part of the prevention team. I’m on the Adult Violence Prevention Team [EPVA]. So, I should be more, but it is like… it doesn’t work very well, because we don’t have any time to dedicate to the team. Everything is very, it’s only me and a social worker, and it’s all made in a hurry.”* (Participant 17, Woman)

A few doctors also considered that presenting a report was not their responsibility. This position was essentially assumed by the doctors that disagree with mandatory reporting.

*“(…) I think that we as doctors should not allow being pushed yet another responsibility, that it is not only ours, as I said it is mine as a person, but I don’t think it is mine as a doctor.”* (Participant 08, Woman)

Physical violence was perceived by some doctors as an acceptable response to a cycle of continuous psychological violence, leading the patient to reach a limit, responding aggressively. In these cases where violence was an acceptable response to violence doctors manifested the belief that reporting would not be the answer, preferring to work with the family to cease all kinds of violence.

*“Saying that a man hit that woman, yes, he did. But why did he hit her? Could it be that she, for example, was not systematically saying bad things about everything he does*?” (Participant 10, Man)

### Barriers related to the victim or aggressor

Different barriers described were related to the victim, the aggressor, and the dynamics of a violent relationship. Doctors manifested a desire to respect the victim’s autonomy supporting her in the decision to make a report. In situations where the victim assumes a proactive stance, working on an escape plan and reflecting on the best moment to abandon the relationship, the doctor takes an expectant position. In these cases, premature reporting done by the doctor could be detrimental putting at risk the victim’s plan.

*“When the person shows that she has a plan, that she is in control of the situation and is taking measures to resolve it. In that case I will support her but I will not interfere.”* (Participant 34, Man)

Doctors’ perception of the degree of violence is a concept that can be seen either as a barrier or a facilitator of reporting. It encompasses the doctor’s evaluation of the gravity of violence suffered by the victim, being that in cases where the violence is considered less serious, the doctor will choose not to report it. This perceived “inferior degree” of violence is frequently linked to forms of psychological abuse.

*“I knew there was, I know there is verbal violence… I suspected there was physical violence although I never had any evidence. In that case, I didn’t report, but I think that we end up not reporting something that isn’t evidently physical.”* (Participant 09, Woman)

Doctors’ perception of the risk incurred by the victim after reporting also impacted their decision in taking action. Doctors reflected on the risk of retaliation and escalation of violence against the victim after a report. This subtheme relates to the idea that the legal response to reporting is not sufficiently protective. During the interviews, doctors recollect several cases where after a report made by the victim, the doctors, or others, the aggressors became more violent.

*“(…) If there is a report it can even aggravate the violence or in more extreme cases originate situations that put at risk the life of the victim*.” (Participant 46, Man)

The fear of retaliation, among other reasons, can justify the lack of collaboration of the victim, before and after a report has been presented to the authorities. Doctors considered that the involvement of the victim and their willingness to abandon the relationship is essential to the success of the legal procedures and in their absence, doctors felt discouraged to report.

*“The victim would go to the emergency department multiple times, but afterwards, since in this case, it was her son [the aggressor], she would end up withdrawing the complaint. So it was very complicated, there were many years of… of difficulties on that point, and talking to the social worker, and following up the case just so she would quit.”* (Participant 12, Woman)

### Facilitators related to the physicians’ activity

Doctors also reflected on multiple facilitators and motivators that lead them to report cases of DV to the authorities. The possibility of presenting an anonymous report was seen as a facilitator for some doctors by contradicting some of the barriers previously presented. However, some doctors recognize that this method of reporting may be less efficient than when the report is assumed by the doctor.

*“The report can be made anonymously. That facilitates. But I don’t know if the report would be taken more seriously if they knew it was a doctor that denounced it.”* (Participant 29, Woman)

It is also important to consider that the anonymity of the report does not mean that the victim would not be informed by the doctors of their decision to report. Some doctors consider it important to notify the victim of their intention to report, so she can take measures to protect herself, while others prefer to safeguard their medical relationship with the victim and would not admit to having been the ones presenting the complaint.

Doctors that agree with mandatory reporting laws stated that the knowledge of the case is the only incentive needed to present a report.

*“I would tell her [the victim] that it is a public crime, and so, I will have to report it. It is my obligation as a citizen and as a doctor to report the situation.”* (Participant 44, Woman)

Some doctors admitted to feeling guilty for not reporting previous cases to the authorities assuming a more proactive stance in following cases. These sentiments arise when the doctors reflect on the risk incurred by the victim in staying in an abusive relationship.

*“I remember clearly that she was a little puzzled with that and, at the time, because of my inexperience, I thought I should have taken some measure concerning that family and that child. And even today I regret that I didn’t do anything.”* (Participant 13, Woman)

Contrary to the most common perception that the response offered after reporting is inefficient, some doctors shared previous positive experiences regarding the resolution of DV cases, which leads them to believe in the efficiency of reporting.

*“I had one patient who was tetraplegic and would tell me that his wife would bite him in the head because it was the only place he could feel it. And, at the time, I called the cops and went in person to present a report to the PSP [police]. That gave me a lot of trouble, they found out it was me, but it had a consequence, the patient started getting domiciliary support. There was surveillance and services were mobilized.”* (Participant 39, Woman)

Doctors admitted to having no doubts regarding presenting a report if they were an eyewitness of the occurrence. Even though this is a strong motivator to report, doctors recognize that they are rarely present when violence takes place, and these events do not accurately reflect the majority of cases of DV encountered in their clinical practice.

*“That’s what I was telling you, if I was a witness that I was in the front line and saw the aggression taking place, in that case, there is no doubt.”* (Participant 36, Woman)

### Facilitators related to the victim or aggressor

Doctors also described facilitators of reports related to the characteristics of the victim, the aggressor, or the context of their relationship. In the case of victims who, because of their age, or physical or mental capacity, are unable to protect themselves, doctors agree that the report would be more readily done. Some doctors also cited that the involvement of a fragile individual would motivate them to present a report even in cases where they were not the “direct” victim of the aggression but a spectator. This was especially true in cases where children lived in a violent environment, observing and normalizing the violence between their parents.

*“If there are children or elderly involved, people that are dependent, bedridden, with physical… or mental deficiencies, that wouldn’t allow them to protect themselves. In those situations, I think we don’t have so many doubts in reporting it.”* (Participant 53, Woman)

Doctors would also be more motivated to report in cases where the victims lack a social or familial network of support, structures perceived by the doctors interviewed as protective to the victims.

*“In cases where the person is isolated, when she doesn’t have more family or anybody who she could ask for help. In those cases you have to help [with the report].”* (Participant 31, Man)

If the victim requests the doctor to present a report most of the barriers discussed previously cease to exist, being this a motivator for the doctor to take action.

*“The victim tells me… that she needs help, she doesn’t dare to do it and she needs help. If so, I would report it*.” (Participant 47, Man)

The careful evaluation of the degree of violence and life-threatening risk is considered essential in the assessment of DV cases. Most doctors agree that in extremely violent cases with severe repercussions to the health and well-being of the victim, as well as her eminent security, they would not hesitate in reporting. However, it is important to address that when questioned most doctors confessed feeling insecure regarding their ability to evaluate DV cases, needing more training.

*“Situation where I feel, but this is so hard sometimes, that the person is really having her life at risk. Right? Or at risk of suffering damage, substantial damage, I think in those cases there would be no doubts. But sometimes it is so difficult to evaluate to which point… the limit.”* (Participant 14, Woman)

### Content analysis

All the doctors interviewed were in agreement with the legislation relating to the facultative reporting of DV cases by the general population. However, regarding the mandatory reporting of DV contemplated by the Portuguese law, and applicable to public workers, including those working for the Portuguese National Health System, doctors were almost divided in their opinion with 29 doctors agreeing with mandatory reporting and 25 disagreeing. Using content analysis, we determined which themes and subthemes were more frequently associated with the agreement, or disagreement, regarding mandatory reporting of DV cases (Table 3). Overall doctors that agree with mandatory reporting cite less frequently barriers to reporting (147 citations) than doctors that disagree (160 citations). In contrast, facilitators of reporting are cited more frequently by doctors that agree with mandatory reporting (63 citations) than doctors that disagree (48 citations).

**Table 3:** Frequency of each theme and subtheme in relation to the agreement or disagreement of each participant concerning mandatory reporting cases of DV by family doctors

*Themes and Subthemes*	*Number of participants N (%)*	
	**Agreement**	**Disagreement**
**Barriers related to the physician’s activity**		
Difficulty of detection	10 (19)	9 (17)
Lack of time and bureaucracies	13 (24)	12 (22)
Lack of knowledge	**19 (35)**	**20 (37)**
Lack of guidelines	4 (7)	3 (6)
Fear of false testimony	10 (19)	6 (11)
Breaking doctor-patient relationship	12 (22)	13 (24)
Professional secrecy	6 (11)	9 (17)
Fear of reproach and retaliation against the doctor	4 (7)	9 (17)
Alternatives perceived as more beneficial	7 (13)	11 (20)
Response perceived as inefficient	**18 (33)**	**18 (33)**
Not being a doctor's responsibility	0 (0)	3 (6)
Violence as an acceptable response to violence	0 (0)	1 (2)
**Barriers related to the victim and/or aggressor**		
Victims autonomy	13 (24)	12 (22)
Degree of violence	6 (11)	4 (7)
Risk of retaliation and escalation of violence	11 (20)	13 (24)
Lack of victims' collaboration	14 (26)	17 (32)
**Facilitators related to the physician’s activity**		
Anonymous report	8 (15)	1 (2)
Knowledge of the case	**12 (22)**	**0 (0)**
Feeling guilty for not reporting	3 (6)	3 (6)
Response perceived as effective	3 (6)	1 (2)
Witnessing of the occurrence	3 (6)	6 (11)
**Facilitators related to the victim and/or aggressor**		
Involvement of a fragile individual	**11 (20)**	**9 (17)**
Lack a social or familial network of support	3 (6)	3 (6)
Victim request	1 (2)	8 (15)
Degree of violence and life-threatening risk	**19 (35)**	**17 (32)**

Reflecting on the barriers encountered by the doctors when pondering a report, we found an interposition between the doctors that agree and disagree with mandatory reporting, regarding the subthemes: “Lack of knowledge” and “Response perceived as inefficient”. This concurrence between barriers, independently of the position regarding the law, expressed by the doctors shows how these are the greatest challenges faced by doctors when dealing with DV cases being almost universally experienced. Doctors that disagree with mandatory reporting, also frequently cited as a barrier the “Lack of victims collaboration”. These doctors put a great emphasis on the disposition of the victim to corroborate their deposition and collaborate with the legal procedures to achieve the desired response after reporting, considering that in the absence of victims’ approval reporting would be ineffective.

The most common facilitator of reporting cited by the doctors was: “Degree of violence and life-threatening risk”, appearing in 19 of the interviews with doctors that agree with mandatory reporting and 17 of the doctors that disagree. Doctors’ perception of an extreme degree of violence that would compromise their patient security is the greatest motivator for reporting, even between doctors that do not agree with mandatory reporting, limiting their intervention to these and other specific cases, such as those involving a fragile individual (*n* = 11 between doctors that agree with mandatory reporting and *n* = 9 between those that disagree). Noticeably, only the doctors that agree with mandatory reporting affirm that the simple “Knowledge of the case” is a sufficient motivator for reporting (*n* = 12 VS *n* = 0).

## Discussion

### Key findings

This study reports the perceptions of family doctors in Portugal regarding the barriers and facilitators for them to present, or not, a report to the authorities of cases of DV violence encountered in their clinical practice. The four themes that emerged from the analysis of the interviews represent the dichotomic position faced by these doctors and exemplify multiple doubts and concerns felt when dealing with DV cases. The current legislation that recognizes DV as a public crime, making reporting mandatory for public workers, in this case, doctors, is seen as a controversial policy. Doctors agree with the extension of the duty to report to the general population but are divided in their opinion regarding mandatory reporting for health professionals. This division arises from the multiple barriers faced when trying to submit a complaint, especially with respect to the protection offered to the victim and the efficiency of law enforcement and juridical responses. Most doctors also admitted to a lack of knowledge necessary to confidently and correctly manage DV cases, expressing many doubts when approaching a potential victim and not knowing how to follow, refer or report these situations. This lack of knowledge is cited as the result of a lack of training and institutional guidelines. It could also be the result of misinformation spread by hierarchy superiors, law enforcement, and even juridical departments. Independently of their position regarding mandatory reporting, most doctors agree that the involvement of a fragile person in the abusive relationship, and the perception of an extreme degree of violence, compromising the victim’s health and wellbeing, would be strong motivators for reporting the case.

### Strengths and limitations

This is the first study that investigates doctors’ agreement with mandatory reporting of DV in association with the barriers and facilitators they encounter when considering to present a report to the authorities. The scarcity of studies on the subject justifies the importance of our findings, which will hopefully serve as a basis for ulterior works and an incentive to debate current practices and policies.

The extension of this qualitative study to a national level allowed the participation of doctors from each of the five RHA’s of continental Portugal, including different perspectives based on multiple social, regional and cultural contexts. This widespread approach enriched our work and is one of the main strengths of this study. However, given that participation in this project was entirely voluntary, it is possible that the findings may not fully represent the views of clinicians with limited interest in discussing topics related to DV.

The use of one-to-one semi-structured interviews was also beneficial since it allowed to investigate with more depth these individual experiences, allowing to emerge sometimes even unexpected themes and subthemes, translating different participants’ experiences.

However, the study presents some limitations. Whether participants had personal lived experience of DV themselves or in their families was not investigated, limiting our understanding of the potential influence of their personal experiences in their opinions and motivations. Furthermore, since the lead author is also a family doctor, this may have influenced the responses from the interviewed participants. The interviewed doctors associated their clinical practice with one of the 5 ARS’s of continental Portugal in the sociodemographic questionnaire, providing data on the geographical location of their workplace. However, it is unclear whether they are working within these entities in the public service, in the private healthcare sector, or in both sectors which is a limitation.

### Comparison with the literature

Our study revealed that family doctors in Portugal are divided on their agreement with mandatory reporting of DV. The majority of doctors agrees with the report, especially in circumstances where the victims are incapable of protecting themselves or their health and life is at risk. However, they expressed doubts and concerns that influence their readiness to present a report to the authorities. This conflict of opinions was also observed in a survey, conducted in the USA with doctors working in the primary and secondary healthcare system in California, a state with mandatory reporting laws [14]. In that study, 86% of the doctors surveyed believed that mandatory reporting increases doctors’ ability to detect and respond to DV cases. However, in this same sample, 56% of the doctors revealed not to comply with mandatory reporting in cases where the victim was against the report. The reasons for this noncompliance with the law were: concerns regarding a possible negative impact of the law on victims’ readiness to look for medical attention, the risk of retaliation by the aggressor, breaking doctors-patient confidentiality, and compromising victims’ autonomy.

The dilemma between reporting, or not, a case to the authorities and the barriers described by the doctors in our study were also present in the literature. A cross-sectional survey conducted in Turkey with primary care professionals showed that the majority of doctors (64.3%) report DV cases encountered in their clinical practice [15]. When the initiative to report was not taken by the doctor, victims were incentivized to do so. When questioned, doctors affirmed that the reasons to choose not to make a report were: the belief that the victim would choose to remain in the abusive relationship, lack of knowledge regarding detection, documentation, and referral of DV cases, and concerns about doctors’ security. These answers mimic our observations.

The first barrier encountered by the doctors in our study was the “Difficulty of detection”. A qualitative study with telephonic interviews conducted in California, revealed that only 42% of victims share the abuse to their doctors [16]. However, the stronger facilitator to the disclosure was the direct questioning by the clinician. By questioning the victim in a direct, non-judgemental way doctors can at least in part overcome this barrier. Nevertheless, studies show that doctors rarely question their patients regarding DV [2]. Particularly, in primary care, less than 15% of patients revealed to have ever been asked about DV [16]. A systematic review examining doctors’ perceived barriers to screening for DV divided them into five categories: personal barriers, resource barriers, perceptions and attitudes, fears, and patient-related barriers [17]. Personal barriers encompassed personal discomfort, personal security concerns, and fear regarding misdiagnosis; Resource barriers included lack of knowledge, time constraints, and lack of an office protocol for addressing DV; Fears, perceptions and attitudes were related to the idea that detection DV was not the doctor responsibility, fear of inciting aggressor retaliation against the victim, and concerns about losing their patient trust; and Patient-related barriers, referred to the idea that victims do not want to address the violence choosing to remain on an abusive relationship. It is interesting to note that the same barriers described in this review and faced by the doctors when addressing the victim are also present in our study, influencing doctors’ posture from the initial inquiry to the decision of presenting a report.

Our study revealed that one of the major barriers faced by the doctors in Portugal was “Lack of knowledge”. A Dutch cross-sectional study conducted with 278 mental health professionals questioned them regarding their perception of their knowledge of DV [18]. It demonstrated that most doctors considered that they lack the necessary knowledge to recognize and support victims of DV. Noticeably, a higher perception of their degree of knowledge did not necessarily correlate to a higher degree of factual knowledge. This observation is similar with our results since some of the interviewed doctors confidently responded to questions based on factually wrong information.

Another major barrier to reporting encountered in our study and expressed by the doctors in Portugal was the perception that the response offered after reporting was ineffective in the protection of the victims and persecution of the aggressors. This viewpoint was shared by doctors in Australia interviewed for a qualitative study regarding child abuse [19]. In that study doctors described barriers similar to the ones found in our study: lack of knowledge, lack of guidelines and information about reporting, fear of breaking doctor-patient confidentiality, and fear of an incorrect diagnosis.

One of the principal facilitators of reporting described by our participants was the “Involvement of a fragile individual” in the abuse. The majority of studies regarding mandatory reporting focus on more particular forms of violence like children or elderly abuse. However, the barriers encountered seem to be the same. A study conducted in New York regarding elderly abuse, in the context of emergency services interactions, revealed that doctors frequently chose not to present a report due to a lack of protocols and training, difficulties contacting social services, lack of time, and lack of feedback after reporting [20].

We also found that doctors in Portugal consider the evaluation of the degree of violence as an integral part of the assessment of DV cases, frequently considering reporting situations where the violence is perceived as more serious and potentially life threatening. This is the approach recommended by the Portuguese General Health Department [21]. The focus on the degree of violence and the position assumed by family doctors in Portugal when faced with more extreme cases, with possible life-threatening consequences, may prevent some tragic conclusions, however, it may be considered insufficient in the majority of cases. Qualitative studies conducted in the Philippines and Peru suggest that women consider psychological abuse more degrading and intolerable than physical violence and the majority of victims of DV seek medical help more frequently as a result of prolonged cycles of intimidation than as a response to an isolated episode of abuse [22]. The threat of more serious forms of violence can be sufficient to compromise the health of the victim. A USA study conducted in Texas investigating the relationship between exposure to firearms and negative health outcomes on DV victims showed that the ownership of a firearm by the aggressor is significantly associated with worse victim’s physical health even in cases where firearm related DV never occurred [23].

Our study focused on the opinions of family doctors regarding mandatory reporting, and what compels them to present or not a report. The success of mandatory reporting laws is linked with the degree of compliance by health care professionals with the legislation. However, studies on the efficiency of mandatory reporting are limited. A USA study based on data from the Los Angeles Sheriff’s Department analysed the number of dispatches for DV in the years prior and after the implementation of mandatory reporting legislation, observing no differences in the number of reports by health care professionals [24]. This was attributed to many reasons: lack of knowledge about the new law, lack of compliance, and deterrence from the victim in divulging the abuse. The barriers and facilitators encountered in or study could provide an additional explanation to the degree of compliance by health care professionals with the law.

The sparsity of studies analysing the efficiency of mandatory reporting laws of DV and the motives that lead health professionals to report, or not, these cases to the authorities, prevents a complete understanding of this kind of legislation and its impact on the lives of victims, aggressors, and individuals faced with the obligation to report.

### Implication of the findings for future practice and research

Our study demonstrates the need to intervene with family doctors in Portugal to improve their response to DV cases. Importantly, most of the barriers found in our study are related to the physician’s activity. This calls for the creation of educational programs developed to answer the barriers encountered and faced by family doctors from detection until reporting DV cases. More training opportunities should be available, and doctors should be allowed to manage their consultations more freely enabling them to allocate more time to oversee not only DV cases but other social issues.

In 2014, the General Directorate for Health (DGS) has published a document addressing the approach, diagnoses and intervention of interpersonal violence by health services [21]. This document needs to be further disseminated among health professionals and can serve as a guide for a more focused and community base intervention. Since primary care in Portugal is organized in regional-based unities, current guidelines should be revalued, and protocols established based on the resources available in each community, namely the accessibility of specialized services, support institutions, and police and juridical services. Each health unit should possess a comprehensive and easy to follow algorithm to facilitate the management and reporting of DV cases. An investment should be made to provide support to organizations, and specifically EPVA’s, groups formed in each health unit to respond to DV, with greater financial and human resources.

The DGS reported an increase in violence against health professionals of 40% in 2022 when compared with the previous year [25]. Resources should be allocated to guarantee health professionals’ security, allowing them to conduct their practice in a safe environment, without fear of retaliation when faced with a DV aggressor.

The apparent conflict between mandatory reporting and doctors-patient confidentiality should also be addressed. According to the DGS doctors-patient confidentiality should only be broken in extreme cases of violence that put at risk the imminent security of the victim. However, the evaluation of the degree of violence is based on the understanding of the doctor of each situation. This approach can be highly subjective, compromising health care services efficiency in responding to DV cases, especially when associated with the lack of training and knowledge cited by the doctors.

To establish an effective response to DV, it is also necessary to understand the phenomenon in all its dimensions. Further research through qualitative studies with both victims and aggressors should be conducted, to investigate their perceptions of their interactions with different services: the health care system, support institutions, law enforcement, and the juridical system. Research that evaluates the experiences and opinions of other professionals, namely doctors with other medical specialities and across diverse clinical sectors, nurses, and law enforcement, should also be considered.

The efficiency of mandatory reporting laws in providing victims protection and persecuting aggressors should be carefully assessed. It is not enough to make the reporting of DV cases mandatory. The success of mandatory reporting laws and the standard of compliance with this kind of legislation depends on the perception of the degree of support and protection offered to the victims. Interventions that focus on the social and juridical responses to DV should be implemented to guaranty the success of the legislation. If, however, after careful study and consideration, mandatory reporting laws fail to provide an efficient and protective response to DV, this legislation should be reconsidered.

## Conclusions

This study investigated the motivations of family doctors in Portugal to present, or not, a report of DV cases to the authorities, and adds knowledge to an under researched subject providing the bases for a wider debate concerning current practices and legislation. The themes and subthemes identified in this study represent several of the international concerns and opinions voiced by healthcare professionals in other parts of the world.

The mitigation and prevention of DV imply a continuous effort on a social and legislative level to answer victims’ necessities efficiently and protectively. The results of this study can help develop new interventions for DV, and the development of new clinical, social and legislative approaches.

### Supplementary Information

**Supplementary Material 1.**

## Data Availability

The anonymised datasets analysed during the current study are not publicly available due to concerns regarding participants’ confidentiality but are available from the corresponding author upon reasonable request.

## Abbreviations

APAV: Portuguese Support Victims Association
DV: Domestic Violence
EPVA: Adult Violence Preventions Teams
GNR: Republican National Guard
PSP: Public Security Police
