# Supplementary material for: Barriers and facilitators of the reporting by family doctors of cases of domestic violence – a qualitative study across Portugal
Source: BMC Prim Care. 2024 Apr 5;25:107. doi: 10.1186/s12875-024-02329-0 (PMC10996166; doi:10.1186/s12875-024-02329-0)

**Supplementary Material**

**Appendix 1** – Interview Guide

**Interview guide**

The following questions serve as a guide for the interviewer during the semi-structured interview. It is optional to follow the order of questioning presented below. The interviewer may reformulate the questions to create a logical occurrence with participants’ answers.

1. **How would you define domestic violence?**

- Different forms of violence – psychological, physical, sexual;
- Individuals involved – between couples, during dating, other family members involved;
- In reference to criminality.

1. **What do you think about the mandatory reporting of domestic violence to the authorities?**

- Implications for the general population;
- Implications for doctors.

1. **In your professional experience, did any of your patients tell you they were a victim of domestic violence?**

(If the doctor did not have direct experience with domestic violence cases, did they know about any cases from their colleagues?)

**3.1. What did you do in that situation?** / What was done in that situation?

**3.2. What difficulties did you encounter?** / What problems were encountered?

**3.3. If it was today, would you have done the same?** / If it was you in that position, would you have done the same?

1. **In your opinion, in what way could the intervention by family doctors have an impact in the life of domestic violence victims?**

- Health;
- Quality of life;
- Recurrence of violent episodes;
- Security.

1. **Which factors would encourage you to report a case of domestic violence to the authorities?**
2. **Which factors would make it more difficult for you to report a case of domestic violence to the authorities?**

**Appendix 2 –** Sociodemographic questionnaire

**Sociodemographic questionnaire**

We invite you to answer these questions to better understand the diversity of the participants in our study. All the information is confidential. Please write your answer in the correct space or select one or more of the available options that better describe you.

**Age**: ________ years

**Sex**: □ Female; □ Male; □ Other. Which? _________________________________________

**How would you describe your sexual orientation**? □ Heterosexual; □ Homosexual;

□ Bisexual; □ Other. Which? ______________________________________________________

**How would you describe your ethnicity**? □ White; □ Black; □ Asian; □ Romani; □ Mixed ethnicity; □ Other.

Which? _______________________________________________________________

**How would you describe your marital status?** □ Single; □ In a relationship without cohabitation; □ In a relationship with cohabitation; □ Married; □ Divorced;

□ Widow(er); □ Other. Which? __________________________________________________

**For how many years have you been working as a family doctor**?_______years

**Currently you are working in a**: □ Big city; □ City; □Village; □ Little village

**In which RHA do you currently work?** ____________________________________________

Please provide us with your **email address** to facilitate further contact and the scheduling of the interview: ____________________________________________________________________

**Appendix 3** – Tables with themes, subthemes and illustrative quotes.

**Table S1:** Barriers related to the physician’s activity

| ***Barriers related to the physician’s activity*** | |
| --- | --- |
| Difficulty of detection | *“It’s different forms of violence. It’s not only who gets battered. Because it’s very evident who gets battered. Ok, that one died because he hit her. But there are other kinds of violence that are* ***not always easy to detect****.”* (Participant 13, Woman) |
|  | *"I don't always succeed,* ***sometimes I may suspect something****, but if the* ***patient doesn't tell me anything****,* ***does not confirm my suspicion****, I'm not always able, or don't have the time, to be attentive to all the signs or remember "Wait, this could be domestic violence.””* (Participant 26, Woman) |
| Lack of time and bureaucracies | *"I also want to say that in family medicine we have everything. What* ***we don't have is the necessary time*** *to deal with all of these things. Because there is an* ***excess of bureaucratic burden****."* (Participant 10, Man) |
|  | ***We have 20 minutes consultations here, now not even that, right?*** *[…] We then have the rest of the people outside waiting for their consultation, which they also have the right to have. It’s a bit complicated for us in 20 minutes, to be able to have a meaningful conversation. And also to follow her [the victim] from week to week, or day to day, according to her needs, it’s also complicated. We have many patients, a long list and little time, right? And we are not just dedicated to that [domestic violence].”* (Participant 17, Woman) |
| Lack of knowledge | *“You can't. You can’t [referring to the reporting of DV cases]. I asked the juridic department. You can’t. I had that problem and you can’t. Because it is the story that that woman is telling you. She could have, she could have had an injury, she could… you don’t know. And since you don’t know, you didn't see [the aggression], you can’t.”* (Participant 11, Woman) |
|  | *"Honestly,* ***I also don’t know exactly which are the procedures****. (...) I have a victim here in front of me, who should I call? Honestly, it is a flaw on my part. I have to look up for info into that later.”* (Participant 19, Man) |
| Lack of guidelines | ***“Lack of action protocols. More immediate, more assertive****. This is what we should do, and this, and this. Not grey areas, in which “it’s better not”, “let’s not ruin the relationship”, “speak with APAV first”, “turn around” and, meanwhile, the victim is not even in this world anymore!”* (Participant 32, Man) |
|  | *“****There should be an algorithm****, something concrete that would tell you, in this case, you must do this, refer to this and that institution, you can present a report through these and that means.”* (Participant 51, Woman) |
| Fear of false testimony | *“Because it is like this, you rarely see any physical marks. Even a belt or something like that… it’s what people tell you (...),* ***we don’t know what is true****, we hear people talking, but we don’t know what is true, and then* ***you have the other side of the story****. Since we are a family doctor we have both components, and then sometimes as well… I’m not a cop! To clarify the situation.* ***And I don’t always believe it****.”* (Participant 39, Woman) |
|  | *“I have other cases, a case of a neighbor, that went and made… not a public declaration, it is a public complaint of domestic violence, to a patient, that is, it would be the boyfriend of my patient, but there was no [violence]. He was only very, expressive when [football team] Benfica played.* ***But in reality, her daughter and her [the alleged victim] didn’t have any complaint.*** *And she [the alleged victim] had to explain that it caused tremendous inconvenience to have the PJ [Police] at home, since their daughter could be removed.”* (Participant 32, Man) |
| Breaking doctor-patient relationship | *“It is complicated to keep the relationship. Because you can never again establish a level of trust if you make a report.* ***Who is gonna trust us?*** ***Neither the aggressor nor the victim****. Especially the relationship with the aggressor is destroyed”.* (Participant 22, Woman) |
|  | *“In reality I agree [with mandatory reporting], of course as a family doctor sometimes it is a little bit complicated, and it can* ***shake a little bit the foundations of doctor-patient relationship****.” (Participant 41, Woman)* |
| Professional secrecy | *“Another issue is* ***professional confidentiality****, isn’t it? It all depends if the victim is willing to present a report, and that isn’t a problem, right? Now if they inform me* ***in confidence*** *that they don't want anyone to know, I have an obligation, by law, as a healthcare professional, to safeguard confidentiality. Here we enter a very sensitive area.”* (Participant 35, Woman) |
|  | *“If the victim tells me something in confidence, I cannot report it.* ***Professional secrecy is also law.*** *You can’t… of course it depends on the situation, but if the victim doesn’t want to I think that I should keep that secrecy.”* (Participant 54,Woman) |
| Fear of retaliation against the doctor | *“I explained everything [a suspected case of sexual violence of a father against his daughter stated in consultation by the mother] to the investigator, to the inspector, and the inspector told me: “You know doctor, everyone says it's not true, and right now you are their accuser.* ***They can take you to court for defamation****.” I was so choked I could die, as you can imagine.”* (Participant 06, Woman) |
|  | *“[…] let’s imagine, that I make a complaint, actually I never had any problem, but* ***I have colleagues that became victims of the aggressor****. Who hit them. That retaliate. Retaliation exists!”* (Participant 39, Woman) |
| Alternatives perceived as more beneficial | *“****Just because victims don’t want to report it doesn’t mean they don’t want help.*** *I think that if the person is not ready to report, then we need to find alternatives. I usually ask APAV for help.* (Participant 28, Woman) |
|  | *“I think that in the case of this family* ***reporting the case will not help****, it will not be helpful for the police to go there, it's not gonna help the family. Maybe me* ***as the family doctor, with the help of the psychologist****, something more familiar, maybe she [the victim] would gain conscience, and he [the aggressor] will realize that he is abusing her and that it is a crime, maybe if I say that and explained that his wife symptoms are related to the abuse, maybe that could help.”* (Participant 45, Woman) |
| Response perceived as inefficient | *“I am part of the prevention team. I’m on the* ***Adult Violence Prevention Team*** *[EPVA]. So, I should be more, but it is like…* ***it doesn’t work very well,*** *because we don’t have any time to dedicate to the team. Everything is very, it’s only me and a social worker, and it’s all made in a hurry.”* (Participant 17, Woman) |
|  | ***“Is the woman really protected? He is punished in an exemplary fashion?*** *How? In what way? Does he learn, does he not? (...) People told me: "Why would I report? The first time I went to the police and they told me: "Lady! For the love of God. We have better things to do! But then why? What is gonna happen? He isn’t going to jail! It's no use to complain.""* (Participant 37, Man) |
| Not being a doctor’s responsibility | *"(...) I think that we as doctors should not allow* ***being pushed yet another responsibility, that it is not only ours****, as I said it is mine as a person, but I don't think it is mine as a doctor."* (Participant 08, Woman) |
|  | *“****I’m not here to make a report****. I think that’s the person’s [the victim] decision. My duty is to treat and support, not to report anyone.”* (Participant 52, Woman) |
| Violence as an acceptable response to violence | *"Saying that a man hit that woman, yes, he did.* ***But why did he hit her?*** *Could it be that she, for example, was not systematically saying bad things about everything he does?"* (Participant 10, Man) |
|  |  |

**Table S2:** Barriers related to the victim or aggressor

| **Barriers related to the victim or aggressor** | |
| --- | --- |
| Victims autonomy | *“If the victim… would show me that* ***she has a plan to get out of the situation****, that she already has a* ***circle of support****, and is waiting for the right timing to be able to leave that situation.”* (Participant 04, Woman) |
|  | *“When the person shows that* ***she has a plan****, that* ***she is in control*** *of the situation and* ***is taking measures to resolve it****. In that case I have to support her but I will not interfere.”* (Participant 34, Man) |
| Degree of violence | *“I knew there was, I know* ***there is verbal violence****… I suspected there was physical violence although I never had any evidence. In that case, I didn’t report,* ***but I think that we end up not reporting something that isn’t evidently physical****.”* (Participant 09, Woman) |
|  | *“Depends on the violence, doesn´t it?* ***Psychological violence is more difficult, more difficult to prove.*** *It is also bad, but… the person is not at risk. You make a report and then what happens? How are you going to prove it? And a person is arrested because it calls names or… it’s not! So, in that case I think it needs to be the person, the victim to want to get out of the situation.”* (Participant 49, Woman) |
| Risk of retaliation and escalation of violence | *“Because it is like this, after the report the aggressor continues to be there, continues to exist, and may even continue to live with the victim. So, if he knows that there was a report, and even worst if he doesn’t know who made it,* ***he can take revenge****. I had cases like this in which after reporting the person got beaten even more.”* (Participant 27, Woman) |
|  | *“(...) If there is a report it can even* ***aggravate the violence*** *or in more extreme cases originate situations that* ***put at risk the life of the victim****.”* (Participant 46, Man) |
| Lack of victims’ collaboration | *“The victim would go to the emergency department multiple times, but afterwards, since in this case, it was her son [the aggressor],* ***she would*** ***end up withdrawing the complaint****. So it was very complicated, there were many years of… of difficulties on that point, and talking to the social worker, and following up the case* ***just so she would quit****.”* (Participant 12, Woman) |
|  | *“[…] the victims either said what happened […] or sometimes devalue the situation. […] That hindrance that causes resistance when the* ***victim devalues what she is saying****, afterward I also take one step back… is this being an exaggeration or not.* (Participant 41, Woman) |
|  |  |
|  |  |

**Table S3:** Facilitators related to the physician’s activity

| **Facilitators related to the physician’s activity** | |
| --- | --- |
| Anonymous report | *“****The report can be made anonymously. That facilitates.*** *But I don’t know if the report would be taken more seriously if they knew it was a doctor that denounced it.”* (Participant 29, Woman) |
|  | *“I think that a report, as far as I know, I didn’t make any, but it doesn’t have to be necessarily… assumed as, it can be* ***an anonymous report*** *and that aspect is a facilitator ”* (Participant 46, Man) |
| Knowledge of the case | *“Not long ago I had training with a judge. And he told us that it was to report.* ***It’s a public crime it’s to report****.”* (Participant 40, Woman) |
|  | *“I would tell her [the victim] that* ***it is a public crime****, and so, I will have to report it.* ***It is my obligation as a citizen and as a doctor to report the situation****.”* (Participant 44, Woman) |
| Feeling guilty for not reporting | *“I remember clearly that she was a little puzzled with that and, at the time, because of my inexperience, I thought I should have taken some measure concerning that family and that child.* ***And even today I regret that I didn’t do anything****.”* (Participant 13, Woman) |
|  | *“Especially for not reporting, right? It’s more often than not that we think about it, if* ***we should have done something else****, isn’t it?”* (Participant 17, Woman) |
| Response perceived as effective | *“I had one patient who was tetraplegic and would tell me that his wife would bite him in the head because it was the only place he could feel it. And, at the time, I called the cops and went in person to present a report to the PSP [police]. That gave me a lot of trouble, they found out it was me, but it had a consequence, the patient started getting domiciliary support.* ***There was surveillance and services were mobilized.”*** *(Participant 39, Woman)* |
|  | *“Until today I only reported one case. But it went well. It took time, but* ***in the end, it went well****, so much that the lady that was not even my patient now wants to be.”* (Participant 40, Woman) |
| Witnessing the occurrence | *“****If someone assaulted a person in front of me****, but in that case, I wouldn’t report as a doctor, right? It wouldn’t be something that someone told me. If I had really seen it would be easier [to report].* (Participant 14, Woman) |
|  | *“That’s what I was saying, if I was* ***a witness,*** *if I was in the front line and* ***saw the aggression taking place****, in that case, there is no doubt.”* (Participant 36, Woman) |
|  |  |

**Table S4:** Facilitators related to the victim or aggressor

| **Facilitators related to the victim or aggressor** | |
| --- | --- |
| Involvement of a fragile individual | *“I can tell that everything that has to do with children, I went to court and all because I report everything. This is, I report everything that has to do with children, either to CPCJ [Commissions for the Protection of Children and Young People] or to the court.”* (Participant 41, Woman) |
|  | *“If there are* ***children or elderly involved, people that are dependent, bedridden, with physical… or mental deficiencies****,* ***that wouldn’t allow them to protect themselves.*** *In those situations, I think we don’t have so many doubts in reporting it.”* (Participant 53, Woman) |
| Lack of social or family support | *“If I identified that it was a person with a* ***poor social or familiar network*** *that didn’t have other alternative… other person of reference to ask for help, no doubt I would act faster.”* (Participant 09, Woman) |
|  | *“In cases where* ***the person is isolated****, when she doesn't have more family or anybody who she could ask for help. In those cases you have to help [with the report].”* (Participant 31, Man) |
| Victim’s request | *“****The victim tells me****… that she needs help, she doesn't dare to do it and she needs help. If so, I would report it.”* (Participant 47, Man) |
|  | *“****If the person [victim] asked me****. If she asked me, I would report it.”* (Participant 50, Man) |
| Degree of violence and life-threatening risk | *“Situation where I feel, but this is so hard sometimes, that the person is really having her* ***life at risk****. Right? Or at risk of suffering damage,* ***substantial damage,*** *I think in those cases there would be no doubts. But sometimes it is so difficult to evaluate to which point… the limit.”* (Participant 14, Woman) |
|  | *“Repeated situations with a high*  ***degree of violence****, that involved knives or guns,* ***things that can kill*** *or cause irreparable damage.”* (Participant 48, Woman) |
|  |  |

**Appendix 4 –** COREQ (Consolidated criteria for Reporting Qualitative research) Checklist

**COREQ (COnsolidated criteria for REporting Qualitative research) Checklist**

A checklist of items that should be included in reports of qualitative research. You must report the page number in your manuscript where you consider each of the items listed in this checklist. If you have not included this information, either revise your manuscript accordingly before submitting or note N/A.


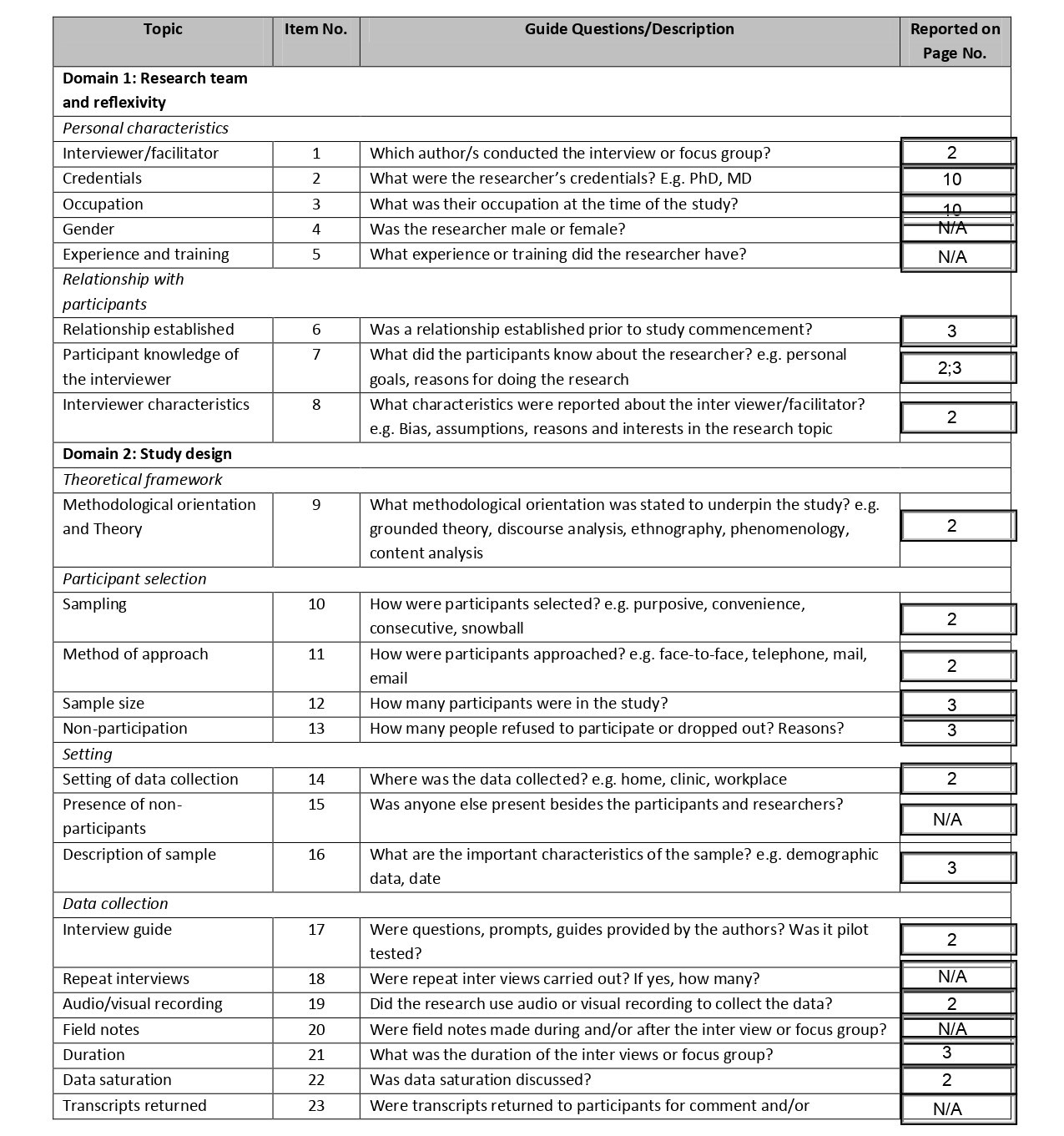


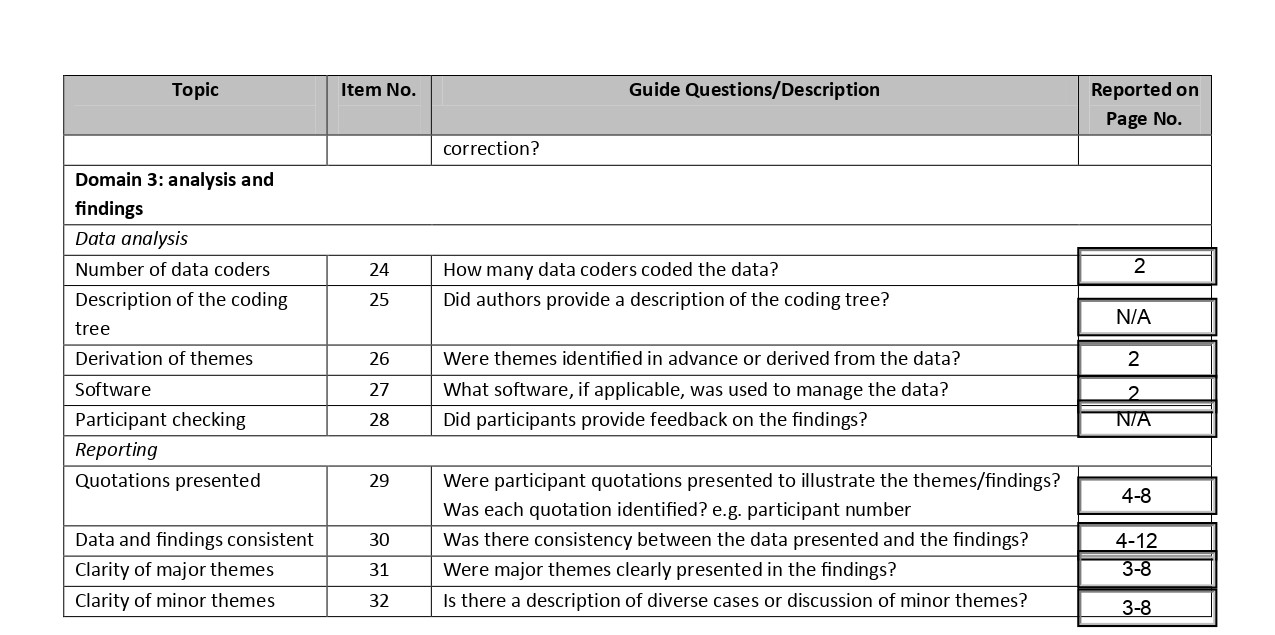

Supplement: Supplementary file 1 — Supplementary Material 1. [file 12875_2024_2329_MOESM1_ESM.docx]
